# A Case of Legionella Pneumonia Suspected as Typical Pneumonia After Lung Cancer Surgery, Diagnosed by a Repeat Urinary Legionella Antigen Test

**DOI:** 10.7759/cureus.98196

**Published:** 2025-11-30

**Authors:** Yusuke Nabe, Hiroshi Mizuuchi, Masaaki Inoue, Junichi Yoshida

**Affiliations:** 1 Department of Chest Surgery, Shimonoseki City Hospital, Yamaguchi, JPN

**Keywords:** acute kidney injury, legionella pneumonia, macrolide therapy, postoperative pneumonia, retesting, urinary antigen test

## Abstract

*Legionella* *pneumophila* is a leading cause of severe atypical pneumonia and is associated with high mortality when initial treatment is inadequate. The urinary *Legionella* antigen test serves as a rapid, first-line diagnostic tool; however, early results may be negative, and antigen shedding can be intermittent. In these situations, timely retesting is essential, especially in postoperative or high-risk patients. We report the case of a 74-year-old man, a former smoker with diabetes and moderate aortic stenosis, who underwent thoracoscopic left lower lobectomy and was discharged on postoperative day (POD) six. He developed a fever on POD 27, accompanied by right upper lobe pneumonia. Outpatient urinary antigen tests for pneumococcus and *Legionella* were negative, and oral garenoxacin was initiated. However, hypoxemia and inflammation worsened, resulting in urgent admission on POD 30. Broad-spectrum β-lactams (sulbactam/ampicillin, followed by piperacillin/tazobactam) were ineffective. Chest CT revealed enlarging infiltrates and a parapneumonic effusion requiring drainage, despite negative pleural fluid cultures. Given the persistent deterioration of the patient's condition, levofloxacin was added, and the urinary *Legionella* antigen test (R70829, MIZUHO MEDY Co., Ltd., Tosu, Japan) was repeated on hospital day five (POD 35), which returned positive, confirming *Legionella* pneumonia. Subsequent targeted therapy with azithromycin (days 6-8 and 13-15), alongside levofloxacin, resulted in clinical improvement. Despite complications during the clinical course, including acute kidney injury necessitating two hemodialysis sessions and a creatine kinase peak of 1,563 U/L, the chest drain was removed on day 21, oxygen was discontinued by day 31, and the patient was transferred to long-term care on day 54. The sequence of renal failure preceding creatine kinase elevation suggests direct renal involvement by *L. pneumophila* rather than primary rhabdomyolysis. Vigilant retesting enabled timely pathogen-directed therapy and a favorable outcome. This case report highlights that a single negative urinary antigen test does not rule out *Legionella* infection. We accordingly recommend early repeat antigen testing, ideally within one week, for postoperative pneumonia unresponsive to β-lactams, together with prompt macrolide or fluoroquinolone therapy.

## Introduction

A prevalence of atypical pneumonia of 8.1% has been reported, with the estimated prevalence of *Legionella* in patients with severe pneumonia at 4.0% (95% confidence interval: 2.8%-5.3%) [1]. Patient factors that increase the risk of developing a *Legionella* infection include male gender, age over 50, smoking, surgery, intubation, nasogastric tube use, ventilator management, and an immunocompromised state (post-organ transplant, cancer treatment, steroid use) [2]. Most cases of Legionnaires' pneumonia are caused by *Legionella pneumophila* serotype one, with the mortality rate estimated to be approximately 27% if not treated appropriately [3]. It has been reported that if postoperative pneumonia is resistant to standard antibiotics or if the symptoms and blood test results are atypical of usual pneumonia, it is extremely important to consider the possibility of atypical pneumonia [2].

We encountered a case in which early retesting of the urinary *Legionella* antigen saved the life of a patient with *Legionella* pneumonia. We report this case to highlight the importance of early retesting in situations where atypical pneumonia cannot be ruled out, even if the initial test is negative.

## Case presentation

**Figure 1:**
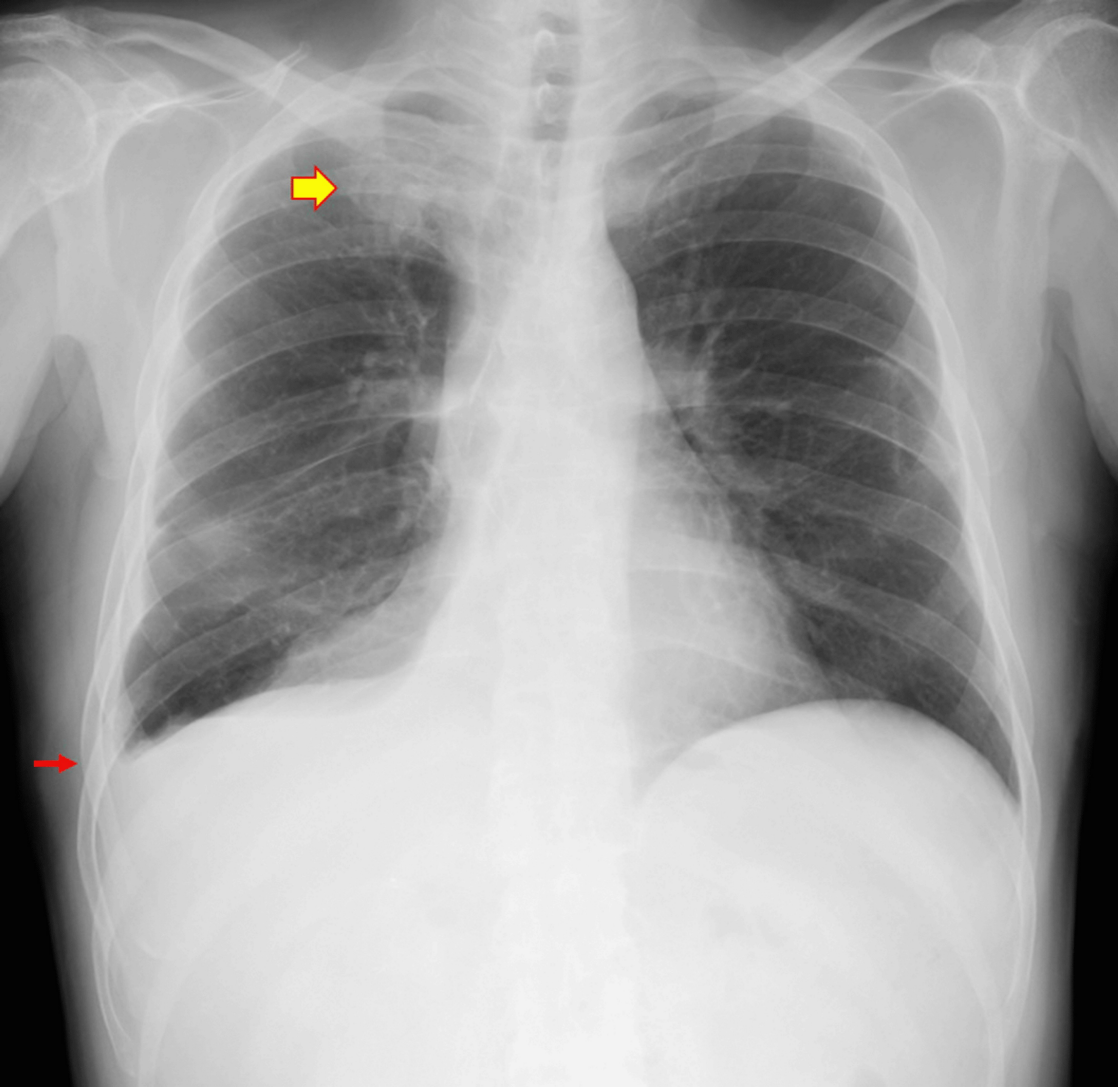
X-ray findings at the time of the onset of Legionnaires' disease (POD 28) An infiltrative shadow appeared in the right upper lung field. Right pleural effusion was noted, consistent with findings following right lower lobectomy. Infiltrative shadow (yellow arrow); pleural effusion (red arrow).

**Figure 2:**
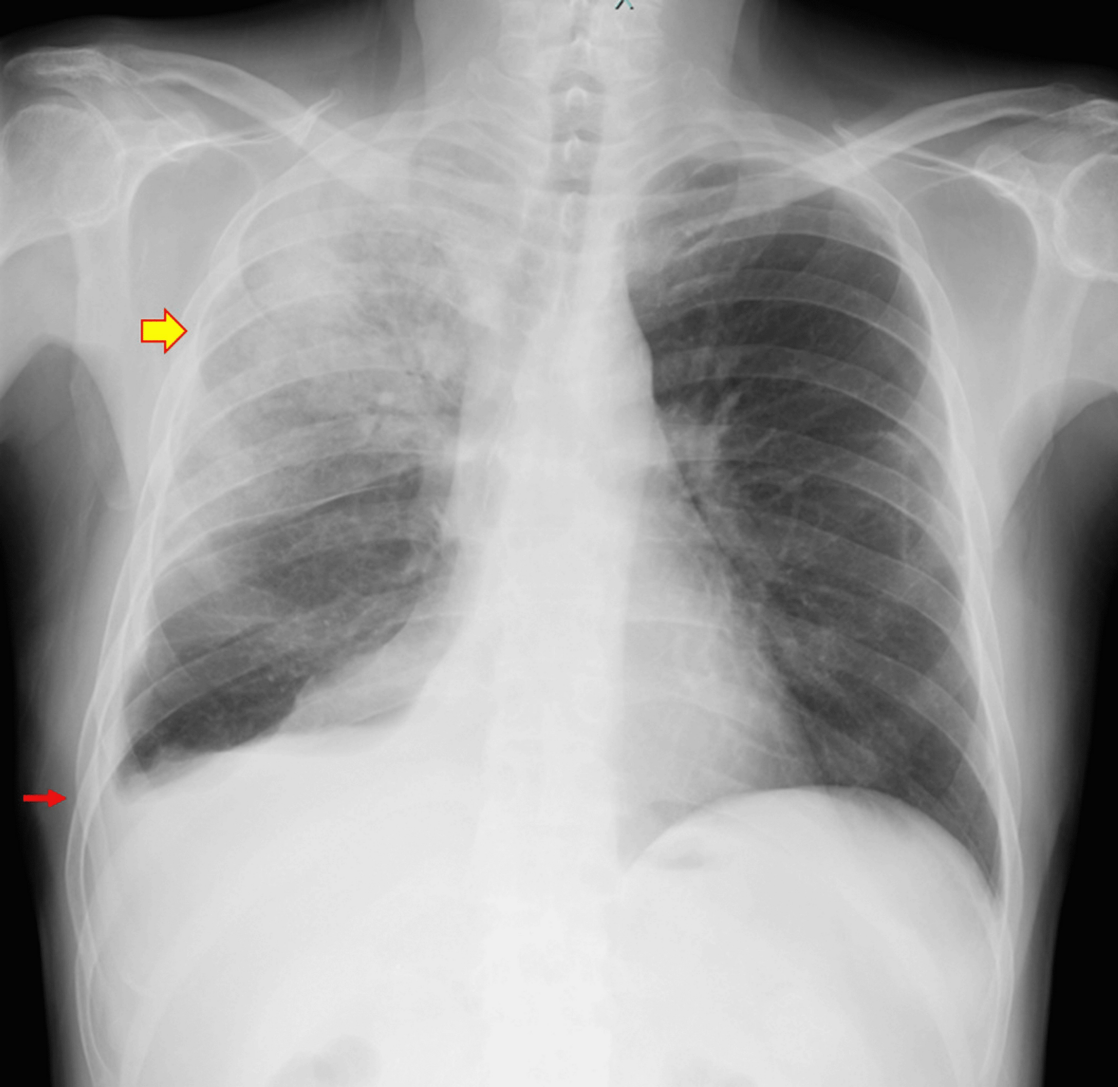
Before right thoracic drainage (POD 32) The infiltrative shadow expanded throughout the entire right upper lung field. An increase in right pleural effusion was suspected. Enlarging infiltrate (yellow arrow); increased pleural effusion (red arrow).

A 74-year-old man with a history of smoking presented with fever. He had previously undergone thoracoscopic partial resection of the left lower lobe for left lower lobe lung adenocarcinoma (pathological stage 0) and was discharged on postoperative day (POD) six with a favorable outcome (no complications). However, on POD 27, he developed a fever and presented to our department on POD 28. He was diagnosed with right upper lobe pneumonia and started on oral treatment with garenoxacin (Figure 1). On POD 30, his pneumonia worsened, and he was urgently admitted to our hospital (Figure 2). His past medical history included pathological-stage IB right lower lobe squamous cell carcinoma of the lung (he underwent right lower lobectomy and mediastinal lymph node dissection five years and two months earlier; there were no postoperative adverse events, and he was discharged on POD six), diabetes mellitus, and moderate aortic stenosis. After surgery for squamous cell carcinoma of the right lower lobe of the lung, the patient received oral UFT for two years as postoperative adjuvant chemotherapy. Subsequent periodic CT scans showed no evidence of recurrence.

Table 1 displays the blood test results at the time of admission. Both influenza and coronavirus tests were negative. Although β-D-glucan was elevated at 35.6 pg/mL, serum *Aspergillus* and *Cryptococcus* antigens were negative. Serum *Mycobacterium avium* complex antibody was negative, and TSPOT.TB was indeterminate. Pleural fluid TB/PCR was negative, and pleural fluid acid-fast bacillus culture was also negative. Urinary antigen tests performed in the outpatient clinic the day before admission were negative for both pneumococcus and *Legionella*.

**Table 1:** Blood test results WBC, white blood cell count; SEG, segmented neutrophils; EOS, eosinophils; BASO, basophils; MON, monocytes; LYM, lymphocytes; CRP, C-reactive protein; LDH, lactate dehydrogenase; AST, aspartate aminotransferase; ALT, alanine aminotransferase; BUN, blood urea nitrogen; Cre, creatinine; Na+, sodium; K+, potassium; Cl-, chloride.

Parameters	Test Results (Normal Range)	Unit
White blood cell (WBC) count	9980 (3300–8600)	/µL
Segmented cell (SEG)	85.2 (50–70)	%
Eosinophil (EOS)	0 (0–4)	%
Basophil (BASO)	0.2 (0–2)	%
Monocyte (MON)	6.6 (4–10)	%
Lymphocyte (LYM)	8.0 (25–49)	%
C-reactive protein (CRP)	21.12 (0–0.14)	mg/dL
Lactate dehydrogenase (LDH)	253 (124–222)	U/L
Aspartate aminotransferase (AST)	32 (13–30)	U/L
Alanine aminotransferase (ALT)	9 (10–42)	U/L
Blood urea nitrogen (BUN)	19.5 (8–20)	mg/dL
Creatinine (Cre)	1.19 (0.65–1.07)	mg/dL
Sodium (Na^+^)	137 (138–145)	mmol/L
Potassium (K^+^)	3.7 (3.6–4.8)	mmol/L
Chloride (Cl^-^)	101 (101–108)	mmol/L

**Figure 3:**
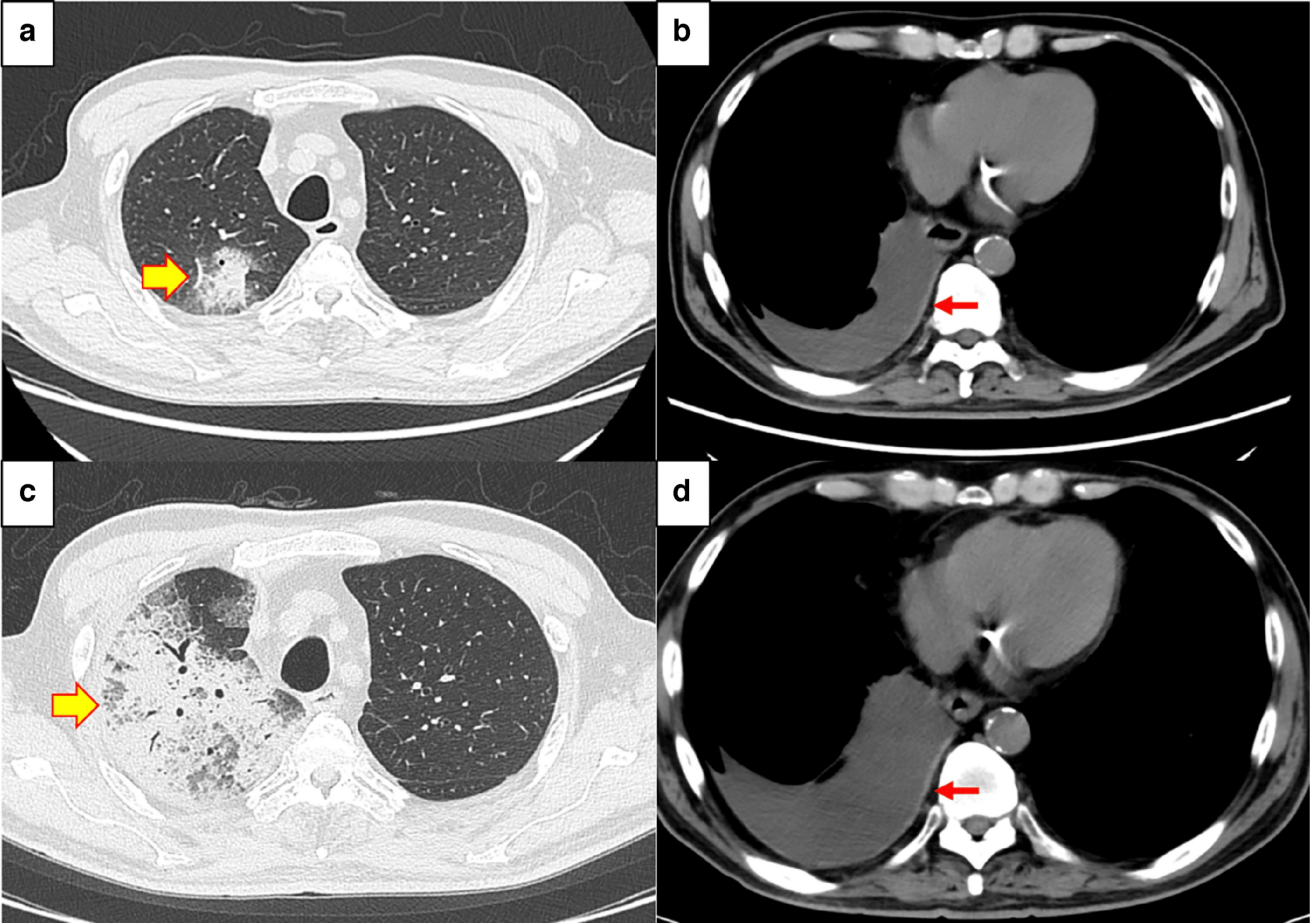
Chest CT findings (treatment progress) a. On POD 28, the infiltrate was limited to a small area within the right upper lobe. Infiltrative shadow (yellow arrow). b. On POD 28, right pleural effusion was observed. The patient had undergone a right lower lobectomy for right lower lobe lung cancer five years and two months earlier, and the finding was thought to be due to the effects of the resection. Pleural effusion (red arrow). c. On POD 32, the infiltrate expanded to cover the entire right upper lobe. Enlarging infiltrate (yellow arrow). d. An increase in right pleural effusion was noted. Increased pleural effusion (red arrow).

After admission, sulbactam/ampicillin was administered from day one to day three; however, the fever persisted, and the inflammatory response significantly increased. Therefore, the antibiotics were switched to tazobactam/piperacillin on day three. Additionally, chest CT revealed an increased right pleural effusion accompanied by hypoxia. The CT scan did not reveal any lesions that suggested a recurrence of lung cancer. Right pleural effusion drainage was performed for both diagnostic and therapeutic purposes (Figure 3).

The pleural fluid pH was 7.2, lactate dehydrogenase 246 U/L, albumin 2.0 g/dL, glucose 200 mg/dL, and total protein 3.1 g/dL. Pleural fluid culture showed no bacterial growth, and pleural fluid cytology revealed no malignant cells. On day four, blood tests showed rising infective markers, and a CT of the thorax confirmed worsening right upper lobe consolidation along with worsening hypoxia. Since Legionnaires' pneumonia could not be ruled out, levofloxacin was added to the regimen. Urinary Legionella antigen (R70829, MIZUHO MEDY Co., Ltd., Tosu, Japan) was measured again on day five and returned positive, confirming the diagnosis of Legionnaires' pneumonia. Azithromycin was administered concomitantly on days 6-8 and 13-15. Oxygen was adjusted to 1-5 L to maintain SpO2 above 90%. In this case, pneumonia was widespread, and we set the target oxygen saturation lower than usual, fearing that high oxygen concentrations could cause interstitial pneumonia or hypercapnia. The treatment course is summarized in Table 2. The patient had been experiencing renal dysfunction since admission and was receiving 1000 mL of intravenous fluid. Fluid management was based on daily blood and weight measurements. As renal dysfunction progressed, the patient underwent two hemodialysis sessions at our hospital's nephrology department. CK levels began to rise on the fourth day after the start of hemodialysis, peaking at 1,563 U/L on the sixth day.

**Table 2:** Treatment process ●: Date of test or medication administration ▲: Date when the peak value of each test was observed AZM, azithromycin; BT, body temperature; CAM, clarithromycin; Cre, creatinine; CRP, C-reactive protein; GRNX, garenoxacin; LVFX, levofloxacin; POD, postoperative day; SBT/ABPC, sulbactam/ampicillin; TAZ/PIPC, tazobactam/piperacillin; WBC, white blood cell

Length of Hospitalization					●	●	●	●	●	●	●	●	●	●	●	●	●	●	●	●	●	●	●	●	●	●	●	●	●	●	●	●	●	●	●	●	●
History of antibiotic use	GRNX		●	●	●																																
	SBT/ABPC				●	●	●																														
	TAZ/PIPC						●	●	●	●																											
	LVFX							●	●	●	●	●	●	●	●	●	●	●	●	●	●	●	●	●	●	●	●	●	●	●	●	●	●	●	●	●	
	AZM									●	●	●					●	●	●																		
	CAM																										●	●	●	●	●	●	●	●	●	●	●
Treatment progress	Fever (37.5°C or higher)	●	●	●	●	●	●	●	●	●	●	●	●	●	●	●	●	●	●	●	●	●	●	●	●	●	●										
	Oxygen administration						●	●	●	●	●	●	●	●	●	●	●	●	●	●	●	●	●	●	●	●	●	●	●	●	●	●	●	●	●		
	Right chest drainage						●	●	●	●	●	●	●	●	●	●	●	●	●	●	●	●	●	●	●												
	Urinary Legionella antigen test		●						●																												
	Hemodialysis												●		●																						
Test results	BT peak 40.2 ℃						▲																														
	CRP peak 73.89 mg/dL									▲																											
	WBC peak 20180/µL							▲																													
	Cre peak 6.32 mg/dL											▲																									
POD	-	27	28	29	30	31	32	33	34	35	36	37	38	39	40	41	42	43	44	45	46	47	48	49	50	51	52	53	54	55	56	57	58	59	60	61	62
Day	-				1	2	3	4	5	6	7	8	9	10	11	12	13	14	15	16	17	18	19	20	21	22	23	24	25	26	27	28	29	30	31	32	33

*Legionella* urinary antigen (R70829, MIZUHO MEDY Co., Ltd.) testing was performed twice, with the second test returning positive. The patient's temperature peaked on the third day of hospitalization and subsided by day 24. Oxygen administration was discontinued on day 32. After the initiation of levofloxacin, the WBC count decreased from its peak. Hemodialysis was performed twice.

**Figure 4:**
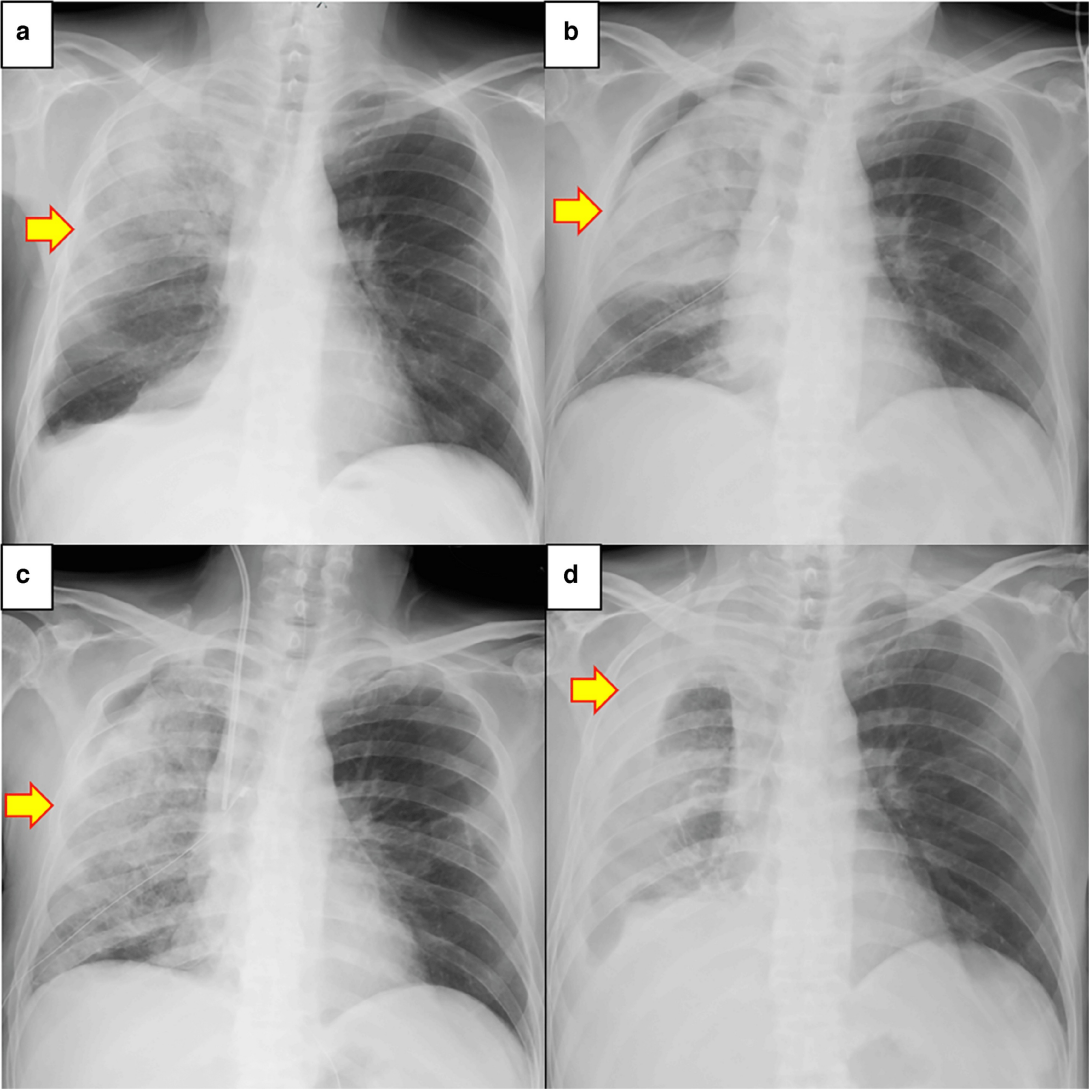
Chest X-ray findings (post-treatment progress) a. POD 32: X-ray findings before right thoracic drainage. An infiltrate was observed in the right upper lung field. Infiltrative shadow (yellow arrow). b. POD 34: Findings after right thoracic drainage. The density of the opacities in the right upper lung field increased. Pleural fluid decreased due to the drainage. Increased density of the infiltrate was observed (yellow arrow). c. POD 42: The infiltrate expanded widely in the right lung, but the infiltrate in the right upper lung field began to improve. The infiltrate shadow expanded throughout the right lung, and the shadow density in the right upper lung field decreased (yellow arrow). d. POD 78: The right pleural fluid increased reactively, but the infiltrate in the lung field tended to improve. No cardiomegaly or left pleural effusion was observed. No recurrence of infiltrate (yellow arrow).

**Figure 5:**
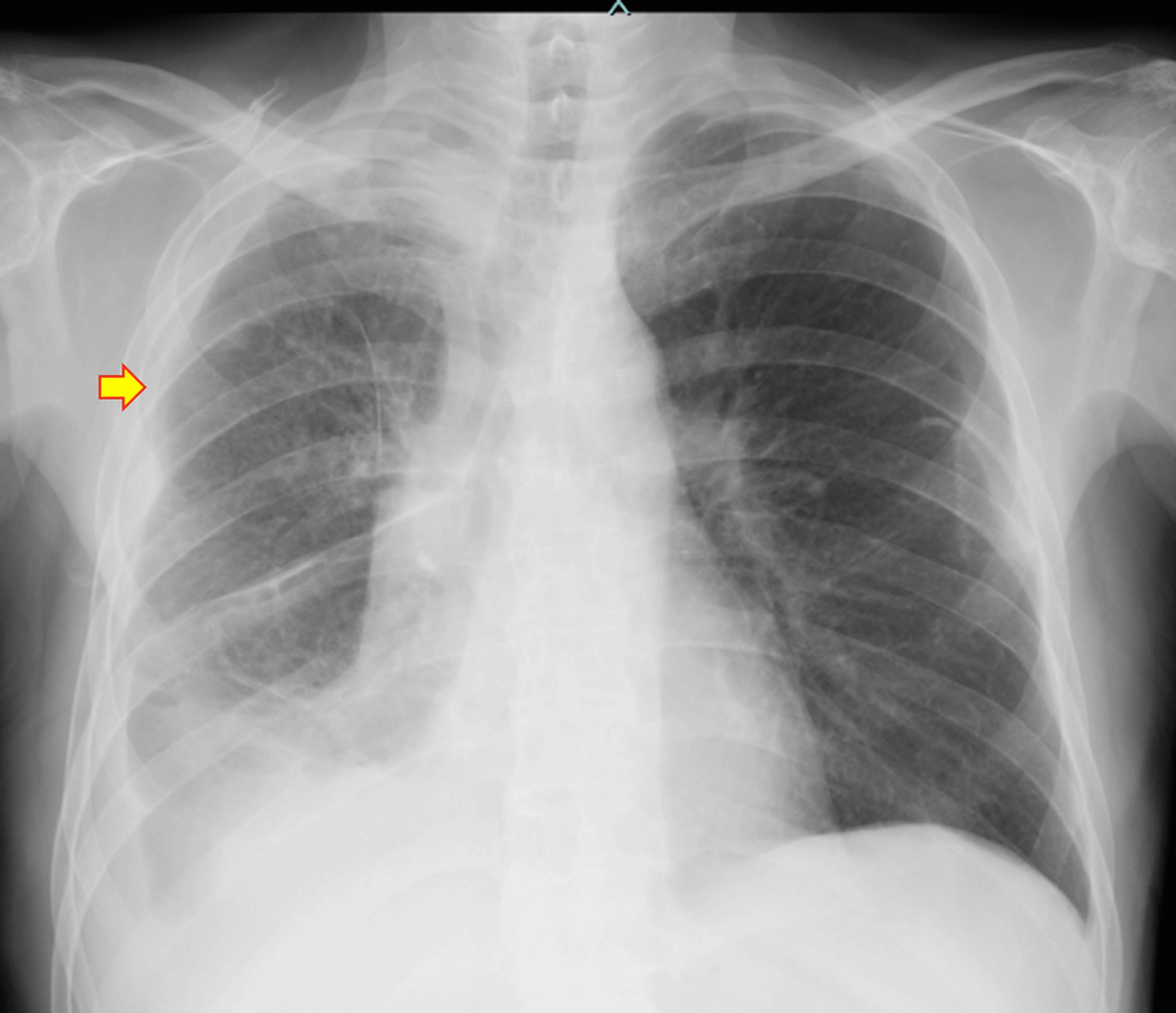
Chest X-ray four months after treatment The infiltrate in the right lung field had significantly improved, and the right pleural effusion had decreased. No recurrence of infiltrates (yellow arrow).

Bacterial culture of the pleural fluid was negative, and the patient was diagnosed with parapneumonic pleural effusion. The right chest drain was removed on day 21. Oxygen therapy was discontinued on day 31, and the patient was transferred to a long-term care hospital on day 54. Chest X-ray findings during the treatment course and the most recent chest X-ray are shown in Figures 4, 5.

## Discussion

This patient developed Legionnaires' disease 27 days after surgery. The initial urinary Legionnaires' antigen test was negative, and the patient was diagnosed with community-acquired pneumonia, with treatment initiated. However, continuing broad-spectrum antibiotics targeting typical pneumonia proved ineffective, and atypical pneumonia could not be ruled out based on the clinical course. Therefore, urinary *Legionella* antigen testing was repeated, and the results were positive, confirming the diagnosis of Legionnaires' disease.

Although no clear environmental factors associated with Legionnaires' disease were identified in this case, known risk factors include male gender, age over 50, smoking, and the effects of surgery [2]. Antibiotics remain the cornerstone of treatment, with macrolides and fluoroquinolones being the recommended drug classes [4].

Legionnaires' disease can lead to serious extrapulmonary complications, including acute kidney injury (AKI), rhabdomyolysis, and damage to the heart and liver, highlighting the importance of rapid diagnosis and prompt treatment [5]. A previous case report describes a patient with renal dysfunction due to *Legionella* infection who required intermittent hemodialysis for 16 days during hospitalization and was discharged 23 days after admission [6].

In our case, renal function improved following two hemodialysis sessions. There are two hypotheses regarding the pathogenesis of AKI: immune-related and infection-related mechanisms. Some suggest that AKI may be indirectly related to *Legionella pneumophila* via rhabdomyolysis, while others propose that it may be directly caused by the pathogen itself [7].

Kidney biopsies from patients with *Legionella* infection have revealed a wide range of cytopathological findings, from tubulointerstitial nephritis (TIN) to acute tubular necrosis (ATN) to glomerulonephritis [8]. Hemodialysis is required in 55.5% of cases of acute renal failure associated with Legionnaires' disease, and the mortality rate is reported to reach 51% (compared with 15% in patients without signs of acute renal failure) [7]. In cases of tubulointerstitial nephritis, steroids have been shown to be effective in restoring kidney function [8]. In this case, CK levels began to rise on the fourth day after the start of dialysis and peaked on the sixth day. The increase in CK levels after renal dysfunction began suggests that *Legionella pneumophila* itself may be directly involved in the renal dysfunction, rather than rhabdomyolysis being the primary cause.

Although a renal biopsy was not performed in this case and steroid treatment was not administered, renal function improved with hemodialysis and appropriate antibiotic treatment. Urinary antigen tests primarily target lipopolysaccharide present in the cell wall of *Legionella pneumophila* and are widely used as first-line screening methods due to their speed, low cost, relatively simple procedures, and ease of specimen collection [9].

Approximately 8% of patients with Legionnaires' disease do not excrete antigens in their urine [10], and approximately 60% excrete antigens intermittently [11]. Therefore, a negative urinary antigen test does not rule out *Legionella* infection, and retesting is recommended when necessary [9]. The overall mortality rate for Legionnaires' disease is 10%, increasing to 27% in patients who are not initially treated with erythromycin [12].

During the course of the disease, the patient developed renal failure and underwent temporary dialysis. However, by re-measuring urinary *Legionella* antigen early and providing appropriate treatment, the patient was able to discontinue dialysis and ultimately survive. This patient had a history of heart disease (aortic valve stenosis) following lung cancer surgery, placing him at very high risk for reduced pulmonary reserve and worsening heart failure. However, early diagnosis saved his life.

When antibiotic treatment for typical pneumonia is ineffective and imaging findings cannot rule out atypical pneumonia, we recommend re-measuring urinary *Legionella* antigen within at least one week.

## Conclusions

We present a case in which typical pneumonia was initially suspected after lung cancer surgery, but a retest for urinary *Legionella* antigen revealed *Legionella* pneumonia, leading to lifesaving treatment. If antibiotic treatment is ineffective and imaging findings suggest *Legionella* pneumonia, retesting is necessary, even if the initial urinary *Legionella* antigen test is negative.
